# Effectiveness of interventions targeting physical activity, nutrition and healthy weight for university and college students: a systematic review and meta-analysis

**DOI:** 10.1186/s12966-015-0203-7

**Published:** 2015-04-01

**Authors:** Ronald C Plotnikoff, Sarah A Costigan, Rebecca L Williams, Melinda J Hutchesson, Sarah G Kennedy, Sara L Robards, Jennifer Allen, Clare E Collins, Robin Callister, John Germov

**Affiliations:** Priority Research Centre for Physical Activity and Nutrition, University of Newcastle, Callaghan Campus, Newcastle, NSW Australia; School of Education, Faculty of Education and Arts, University of Newcastle, Callaghan Campus, Newcastle, NSW Australia; School of Health Sciences, Faculty of Health, University of Newcastle, Callaghan Campus, Newcastle, NSW Australia; School of Biomedical Sciences and Pharmacy, Faculty of Health, University of Newcastle, Callaghan Campus, Newcastle, NSW Australia; School of Humanities and Social Science, Faculty of Education and Arts, University of Newcastle, Callaghan Campus, Newcastle, NSW Australia

**Keywords:** University, College, Tertiary education institutions, University students, Health promotion, Health behavior, Healthy universities, Physical activity, Exercise, Diet, Nutrition, Weight loss

## Abstract

To examine the effectiveness of interventions aimed at improving physical activity, diet, and/or weight-related behaviors amongst university/college students. Five online databases were searched (January 1970 to April 2014). Experimental study designs were eligible for inclusion. Data extraction was performed by one reviewer using a standardized form developed by the researchers and checked by a second reviewer. Data were described in a narrative synthesis and meta-analyses were conducted when appropriate. Study quality was also established. Forty-one studies were included; of these, 34 reported significant improvements in one of the key outcomes. Of the studies examining physical activity 18/29 yielded significant results, with meta-analysis demonstrating significant increases in moderate physical activity in intervention groups compared to control. Of the studies examining nutrition, 12/24 reported significantly improved outcomes; only 4/12 assessing weight loss outcomes found significant weight reduction. This appears to be the first systematic review of physical activity, diet and weight loss interventions targeting university and college students. Tertiary institutions are appropriate settings for implementing and evaluating lifestyle interventions, however more research is needed to improve such strategies.

**Electronic supplementary material:**

The online version of this article (doi:10.1186/s12966-015-0203-7) contains supplementary material, which is available to authorized users.

## Introduction

Physical inactivity and poor dietary-intake are related behaviors that impact on health and wellbeing and the maintenance of a healthy weight. These behaviors underpin risk of lifestyle related non-communicable conditions [1]. Risk for ischaemic heart disease, stroke, type two diabetes, osteoporosis, various cancers and depression are linked by behavioral and biomedical health determinants such as physical inactivity, poor dietary behaviors and overweight/obesity [2].

The health benefits of engaging in regular physical activity are well established for adults [3]. Strategies to promote physical activity have become an important public health approach for the prevention of chronic diseases [4]. The prevalence of achieving physical activity recommendations declines rapidly between the ages of 18 and 24 [5] when many young people are undertaking tertiary education [6-8]. For instance, in the United States nearly half of all university students are not achieving recommended levels of physical activity [9]. Australian data in the ≥18 year age group indicate 66.9% are sedentary or have low levels of physical activity during 2011-2012 [10]. Similarly in the United Kingdom, 73% of male and 79% of female university students do not meet physical activity guidelines [6]. Further, Irwin [8] suggests that students living on campus are less likely to be active, and thus may be at greater risk of poor health.

Dietary intake patterns that align with national dietary guidelines are associated with reduced risk of developing chronic conditions [11,12], however recent research suggests tertiary students do not achieve these guidelines [13-15]. For instance, in the United States, university and college students have sub-optimal dietary habits compared to such recommendations [16]. Similarly, Australian tertiary students fail to consume the recommended daily servings of fruit (50%) and vegetables (90%) [2]. While students from the UK fail to consume the recommended daily intake of fruit and vegetables (88.7% and 83.5%, respectively) [17].

Commencing college/university is often associated with students having more autonomy over their dietary choices (e.g., food purchasing and preparation). Due to life stage, students may not consider the risk of developing chronic diseases when making food choices [18]. Specifically, factors such as cost, skipping meals, inadequate variety of foods, snacking, and frequent consumption of fast foods may increase students’ risk of poor health [19]. Indeed, studies have reported that considerable weight gain occurs during college/university [20,21]. The associated food selection skills and habits have long-term health impacts [22]. Further, within US institutions a great proportion of freshmen (first year) live in college resident halls, which provide commercially prepared food, take-away and pre-prepared meals. This environment may further contribute to subsequent poor food purchasing and preparation behaviors. Along with these dietary behaviors, physical activity participation also declines in university and college students, which may be due to increased sedentary time when studying and during examination periods [23].

Given the lack of physical activity and healthy eating it is not surprising that the prevalence of overweight/obesity has reached epidemic proportions in young adults. In the USA, the age range of greatest increase in obesity (7.1% to 12.1%) is among young adults aged 18–29 years [7]. Indeed, late adolescence and early adulthood appear to be significant periods of transition, highlighting the importance of understanding factors such as attitudes towards and knowledge of health benefits, as these may be associated with physical activity levels, dietary behavior and obesity prevalence [24]. Improvements to lifestyle behaviors can reduce or prevent the occurrence of non-communicable diseases; therefore strategies to foster healthier lifestyles in the working age population are essential.

Higher education institutions are an appropriate setting to promote healthy lifestyles. First, universities and colleges have the potential to engage large numbers of students in behavior change interventions, and the estimated number of individuals participating in higher education is continuing to rise [25]. It is projected that student numbers in American colleges will reach 22 million in 2014, and that the number of students enrolled in higher education worldwide will reach 262 million by 2025, a marked increase from 178 million in 2010 [26]. Second, higher education institutions have access to a large proportion of students living away from home for the first time, and have the capacity to provide support and establish healthy behavioral patterns that may continue throughout the lifespan. Third, universities and colleges are regarded as organizations that follow high standards of practice which can establish research-based examples for surrounding communities to follow. This allows for the opportunity and responsibility to develop and implement the best available research evidence, and to set a benchmark for other groups to follow [27]. Universities and colleges have a range of facilities, resources and qualified staff, commonly including health professionals, ideal for implementing initiatives to target lifestyle-related health issues. Finally, the possibility that exists for students to deliver initiatives as a part of their study/training to become health professionals adds to the promise for tertiary education institutions as ideal settings for promoting healthy lifestyles.

Evidence suggests that intervention strategies have been successful for students in the higher educational setting [1,5], particularly interventions that seek to empower individuals to achieve their full potential through creating learning and support to improve health, wellbeing and sustainability within the community [28]. In addition, whilst the primary advantage of implementing health promotion programs is to reduce individuals’ health risks, the benefits to higher education institutions in attrition, retention and academic performance are also potential gains [29]. Although a recent review examining the effectiveness of interventions targeting health behaviors (physical activity, nutrition and healthy weight) amongst university staff has been conducted [22], it appears that a review investigating the effectiveness of health behavior interventions on these health behaviors/issues for university students has not yet been performed.

### Objective

The objective of this paper is to systematically review the best available evidence regarding the impact of health behavior interventions to improve physical activity, diet and/or weight outcomes and targeted at students enrolled in tertiary education institutions.

## Review

### Methods

#### Data source

A structured electronic search employing PRISMA reporting guidelines [30] was performed on health-focused intervention studies carried out in tertiary level educational institutions and published between January 1970 and April 2014. MEDLINE with full text, PsychINFO, CINAHL, ERIC and ProQuest were systematically searched [22,31-34]. The following search terms were used: (university OR college) AND (health promotion OR intervention OR program OR education) AND (behavior OR physical activity OR exercise OR diet OR nutrition OR weight). Published articles in peer reviewed journals were considered for the review. Bibliographies of selected studies were also considered. Only manuscripts written in English were considered for the review. Two reviewers independently assessed articles for study inclusion, initially based on the title and abstract. Full texts were then retrieved and assessed for inclusion. A third reviewer was used to make the final decision in the case of discrepancies.

### Study inclusion and exclusion criteria

#### Type of participants

Any study including students attending institutions within the tertiary education sector was included. If other types of participants, e.g., staff were also recruited, only students’ data were extracted.

#### Type of intervention (s)/phenomena of interest

Interventions deemed eligible for inclusion had to be implemented within a tertiary education setting and have the aim of improving physical activity and/or dietary intake and/or weight. Interventions of all lengths were accepted for inclusion within the review.

#### Type of studies

All quantitative study designs (including randomized controlled trials, non-randomized experimental trials, pre-post with no control group) were eligible for inclusion.

#### Type of outcomes

This review considered the following outcome measures specific to the health behavior targeted (an increase in knowledge among participants was not a sufficient outcome):

i) Physical activity related outcomes: steps per day, time spent undertaking vigorous and/or moderate physical activity, VO_2_ max, muscular strength/endurance, energy expenditure, flexibility;
ii) Nutrition outcomes: energy intake, macronutrient composition, core food group consumption, diet quality; and,
iii) Weight related outcomes: weight (kg or lbs), body mass index (kg/m^2^) (BMI), waist circumference (cm), % weight loss, % body fat, waist-to-hip ratio (WHR).

### Risk of bias

Risk of bias was assessed for all included studies by two independent reviewers (SK, SR) (a third reviewer [SC] was consulted and consensus reached in the event of a disagreement) using the Academy of Nutrition and Dietetics Quality Criteria Checklist: Primary Research tool assessing 10 criteria [35]. These criteria included whether: (1) The study clearly stated the research question; (2) If the selection of participants was free from bias; (3) If the study groups were comparable; (4) Description of method of handling withdrawals; (5) Use of blinding; (6) Detailed description of interventions and comparisons; (7) Clear definition of outcomes and valid and reliable measurements; (8) Appropriate statistical analysis; (9) Consideration of limitations; and, (10) Likelihood of bias due to funding. Study quality was classed as *positive* if criteria 2, 3, 6 and 7, as well as one other validity criteria question were scored with a ‘yes’, *neutral* if criteria points 2, 3 6 and 7 did not score a ‘yes’, or *negative* if more than six of the validity criteria questions were answered with a ‘no’.

### Data extraction

Data extraction was performed by two reviewers (SK, SR) using a standardized form developed by the researchers. The data extraction consisted of 11 dimensions; country of origin, target sample and size, participants’ mean age, duration of study, intervention description, participant retention, health behavior, study design, outcomes, results, and significance of results (see Additional file 1: Table S1, Additional file 2: Table S2, and Table 1). Extraction was checked for accuracy and consistency by a third reviewer (SC).

**Table 1:** **Critical appraisal criteria of study methodologies**

**Study**	**Criteria 1**	**Criteria 2**	**Criteria 3**	**Criteria 4**	**Criteria 5**	**Criteria 6**	**Criteria 7**	**Criteria 8**	**Criteria 9**	**Criteria 10**	**Classification**
1. Abu-Moghli et al. 2010 [1]	1	0	0	0	0	0	1	1	1	1	∅
2. Afifi Soweid et al. 2003 [69]	1	0	0	0	0	1	0	0	1	1	−
3. Alpar et al. 2008 [67]	1	0	0	1	0	1	1	1	0	1	∅
4. Bowden et al. 2007 [37]	1	1	0	0	0	1	0	1	1	1	∅
5. Boyle et al. 2011 [38]	1	0	1	0	0	1	0	1	1	1	∅
6. Brown et al. 2011 [39]	1	0	0	0	0	1	1	1	1	1	∅
7. Buscemi et al. 2011 [40]	1	1	1	0	0	1	0	1	1	1	∅
8. Cardinal et al. 2002 [41]	1	0	0	0	0	1	1	1	1	1	∅
9. Cavallo et al. 2012 [19]	1	0	1	0	0	1	1	1	1	1	∅
10. Chen et al. 1989 [42]	1	0	0	0	0	1	0	0	0	1	−
11. Claxton et al. 2009 [43]	1	0	0	1	0	0	1	1	0	1	∅
12. Evans & Mary 2002 [43]	1	0	0	0	0	1	0	1	0	1	−
13. Fischer & Bryant 2008 [45]	1	1	1	0	0	0	1	1	1	1	∅
14. Gieck & Olsen 2007 [46]	1	1	0	0	0	1	1	1	1	1	∅
15. Gow et al. 2010 [47]	0	1	1	1	0	1	1	1	1	1	+
16. Gray et al. 1987 [47]	0	1	0	0	0	0	0	0	1	1	−
17. Grim et al. 2011 [5]	1	0	0	1	0	1	1	1	1	1	∅
18. Ha & Caine-Bish 2009 [49]	1	0	0	0	0	1	1	1	1	1	∅
19. Hager et al. 2012 [50]	1	1	0	0	0	1	1	1	1	1	∅
20. Harvey-Berino et al. 2012 [52]	1	0	0	0	0	1	0	0	1	1	−
21. Hekler et al. 2010 [51]	1	0	0	0	0	1	1	1	1	1	∅
22. Huang et al. 2009 [7]	1	1	1	0	0	1	1	1	1	1	+
23. Ince 2008 [68]	1	1	0	0	0	1	0	1	0	1	∅
24. Kolodinsky et al. 2008 [53]	1	1	0	0	0	0	0	0	1	1	−
25. Lachausse 2012 [54]	1	1	0	0	0	1	1	1	1	1	∅
26. LeCheminant et al. 2011 [55]	1	1	0	0	0	1	1	1	1	1	∅
27. Magoc et al. 2011 [57]	1	1	1	0	0	1	1	1	1	1	+
28. Martens et al. 2012 [40]	1	1	1	0	1	1	0	1	1	1	∅
29. McClary King et al. 2013 [56]	1	0	0	0	0	1	1	1	1	1	∅
30. Musgrave & Thornbury 1976 [59]	0	1	0	0	0	1	0	0	0	1	−
31. Pearce & Cross 2013 [31]	1	0	0	0	0	1	1	1	1	1	∅
32. Pearman et al. 1997 [60]	0	1	0	0	0	0	0	1	1	1	−
33. Peterson et al. 2010 [61]	1	1	0	0	0	1	0	1	1	1	∅
34. Reed et al. 2011 [62]	1	1	0	0	1	1	0	1	1	1	∅
35. Sallis et al. 1999 [63]	1	1	0	0	0	1	1	1	1	1	∅
36. Skar et al. 2011 [70]	1	1	1	1	1	1	0	1	0	1	∅
37. Tully & Cupples 2011 [71]	1	1	0	0	1	1	0	1	1	1	∅
38. Wadsworth et al. [64]	1	1	0	0	0	1	1	1	0	1	∅
39. Werch et al. 2007 [65]	1	1	1	1	0	1	1	1	1	1	+
40. Werch et al. 2008 [73]	1	1	0	1	0	1	1	1	1	1	∅
41. Yakusheva et al. 2011 [66]	1	1	0	0	0	0	0	1	1	1	∅
	37	25	10	7	4	34	23	35	33	41	

Criteria: 1) Was the research question clearly stated? 2) Was the selection of study subjects/patients free from bias? 3) Were study groups comparable? 4) Was the method of handling withdrawals described? 5) Was blinding used to prevent introduction of bias? 6) Were intervention/therapeutic regimens/exposure factor or procedure and any comparison(s) described in detail? Were intervening factors described? 7) Were outcomes clearly defined and the measurements valid and reliable? 8) Was the statistical analysis appropriate for the study design and type of outcome indicators? 9) Were conclusions supported by results with biases and limitations taken into consideration? 10) Is bias due to study’s funding or sponsorship unlikely? ^#^1 = Yes; 0 = No; 0 = Unclear.

MINUS/NEGATIVE (−) If most (six or more) of the answers to the above validity questions are “No”, the report should be designated with a minus (−) symbol on the Evidence Worksheet.

NEUTRAL (∅) If the answers to validity criteria questions 2, 3, 6, and 7 do not indicate that the study is exceptionally strong, the report should be designated with a neutral (∅) symbol on the Evidence Worksheet.

PLUS/POSITIVE (+) If most of the answers to the above validity questions are “Yes” (including criteria 2, 3, 6, 7 and at least one additional “Yes”), the report should be designated with a plus symbol (+) on the Evidence Worksheet.

### Meta-analysis

Results were pooled in meta-analysis if they were available as final values at post-intervention, the number of participants was recorded and interventions were sufficiently similar for comparison. If standard deviations were not available, but other statistics (e.g., 95% CI or standard errors) were available, they were converted according to the calculations outlined in the Cochrane Handbook for Systematic Reviews of Interventions [36]. Heterogeneity was assessed using chi-squared with significant heterogeneity assigned at a *P* value < 0.10. If significant heterogeneity existed, the random effects model was used for statistical analysis; if homogenous, the fixed effect model was used. The data from individual studies on physical activity were combined across studies using standardized mean difference (SMD) due to the differences in reported metrics for total, moderate and vigorous physical activity. A unit conversion was not undertaken. Therefore, the meta-analytical results do not reflect the specific magnitude of effects for each study, but rather the extent to which they are more successful against controls. When a study compared multiple treatment groups with a single control, the sample size of the control was divided equally across the treatment group arms so the participants were not counted more than once in the analysis. All meta-analyses were conducted using Review Manager 5.2; the results were calculated by weighting the amount of information they contribute i.e., the inverse variances of their effect estimates. Intention-to-treat analyses were used where available/reported in the studies. If this information was not available completers analyses was used.

## Results

Overall, 41 studies targeting improvements in student health outcomes (physical activity, diet, weight loss) within tertiary education settings met the inclusion/exclusion criteria. Study characteristics (i.e., country, target sample and size, age, duration, intervention and retention) and risk of bias scores are summarised in Additional file 1: Table S1. Risk of bias assessment indicated eight studies had a negative rating (high risk of bias), 30 were considered neutral, and the remaining four had a positive rating (low risk of bias).

Of the 41 studies identified, 33 were conducted in the United States [5,19,37-66], two in Turkey [67,68] and one each in Jordan [1], Lebanon [69], Scotland [70], Ireland [71], Taiwan [72] and Australia [9]. Study designs included randomized controlled trials (n = 16), non-randomized controlled trials (n = 12) and pre-post designs with no control group (n = 13). Study durations ranged from a 30-minute one-on-one brief motivational intervention [58] to an intervention spanning four academic calendar years [67]. Weight loss was the sole focus in two studies [52,59], physical activity in 11 studies [5,19,41,43,45,46,57,58,63,70,72], and nutrition was the focus of 10 studies [9,39,42,44,48,49,51,53,61,62]. A combination of weight loss and/or physical activity and/or nutrition outcomes were examined by 18 studies [1,37,38,40,47,50,54-56,60,64-69,71,73]. Study participant numbers ranged from 16 [53] to 2971 [50]. Retention rates ranged from 36.6% [46] to 100% [49,51,53,62,71]; five studies [19,42,44,66,68] did not report retention rates.

Three studies did not report the sex distribution of participants [41,57,68]. Of the remaining studies, four had even sex distribution [48,56,62,69], three had a majority of male participants [43,55,61]; 23 studies were comprised of majority female participants [1,5,9,37-40,42,44,47,49,50,52-54,58-60,63,65,70,71,73]; and eight studies consisted of entirely female samples [19,45,46,51,64,66,67,72]. In general, demographic characteristics of participants such as age were not consistently reported.

### Effectiveness of interventions

#### Physical activity and fitness outcomes

As represented in Additional file 2: Table S2, of the 41 studies in our review, 29 examined physical activity (11 exclusively, 18 in combination with other health behaviors). Of these 29 studies, the average risk of bias classification was neutral. Of the studies investigating change in physical activity or fitness behavior, 18 of 29 reported significant improvements from pre- to post-intervention. In five studies a significant increase in physical activity minutes was achieved [50,57,63,70,71]; in five studies the number of days participating in physical activity increased [5,43,58,64,65]; exercise duration increased in three studies [38,65,67]; Metabolic Equivalent of Task (METs) increased in three studies [60,68,72]; exercise barriers decreased in one study [56]; and Physical Activity Readiness Questionnaire (PAR-Q) scores improved in one study [50].

### Meta-analysis

#### Total physical activity

**Figure 1:**
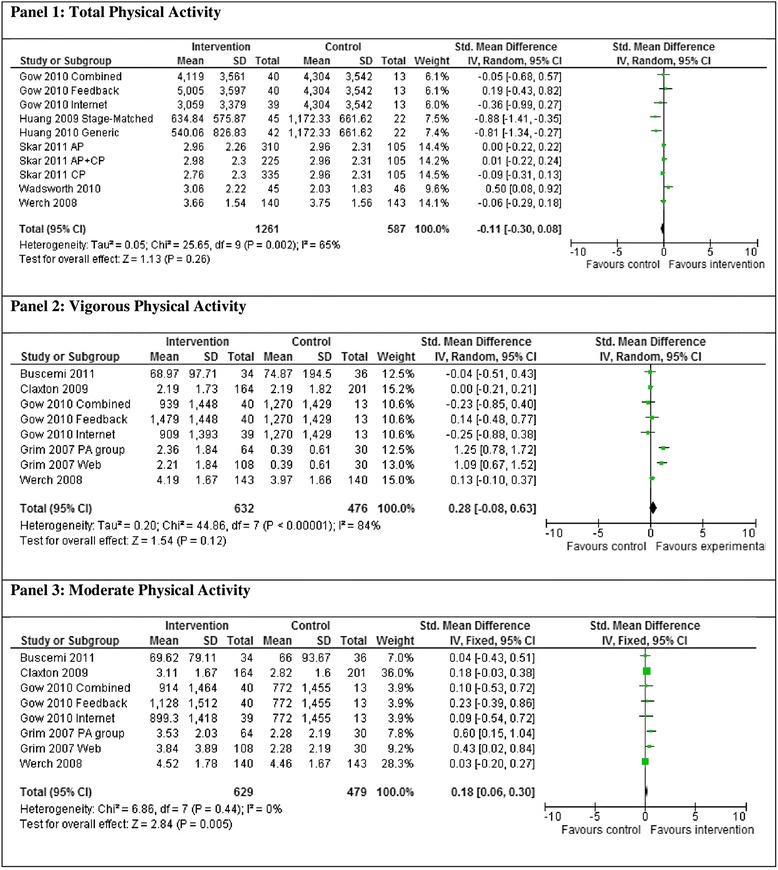
**Meta-analysis of total (panel 1), vigorous (panel 2) and moderate (panel 3) physical activity.**

Five studies comparing interventions targeting health behaviors to a control condition that assessed total physical activity level at post-intervention were combined in meta-analysis (see Figure 1). Three of the studies included multiple intervention arms compared to a single control group, therefore 10, intervention versus control comparisons were included in the analysis.

The studies were significantly heterogeneous (*χ*^2^ = 25.65, d.f. = 9 [*P* = 0.002], *I*^2^ = 65%) and demonstrated no significant difference in total physical activity between intervention and control groups at post-intervention (SMD −0.11 [−0.30,0.08], Z = 1.13 *P* = 0.26).

#### Vigorous physical activity

Five studies comparing interventions targeting health behaviors to a control condition that assessed vigorous physical activity levels at post-intervention were combined in a meta-analysis (see Figure 1). Two of the studies included multiple intervention arms compared to a single control group; therefore eight, intervention versus control comparisons were included in the analysis.

The studies were significantly heterogeneous (*χ*^2^ = 44.86, d.f. = 7 [*P* < 0.001], *I*^2^ = 84%) and demonstrated no significant difference in vigorous physical activity levels between the intervention and control groups at post-intervention (SMD 0.28 [−0.08,0.63], Z = 1.54 *P* = 0.12) (see Figure 1).

#### Moderate physical activity

As represented in Figure 1, five studies comparing interventions targeting health behaviors to a control condition that assessed moderate physical activity levels at post-intervention were combined in meta-analysis. Two of the studies included multiple intervention arms compared to a single control group; therefore eight, intervention versus control comparisons were included in the analysis.

The studies were homogenous (*χ*^2^ = 6.86, d.f. = 7 [*P* = 0.44], *I*^2^ = 0%) and demonstrated significantly greater moderate physical activity levels in the intervention group compared to the control group at post intervention (SMD 0.18 [0.06,0.30], Z = 2.84 *P* = 0.005).

### Nutrition outcomes

Of the 41 included studies, 24 reported nutrition outcomes (10 examined nutrition exclusively), with fruit and vegetable intake the most reported outcome, used in 12 of the reported studies [39,40,44,47,49-51,54,56,62,65,73]. Of the 24 studies, the average risk of bias rating was neutral. Six studies had a negative rating, sixteen were neutral and two had a positive rating.

Interventions were found to be effective in improving nutrition behaviors in 12 of the 24 studies [1,39,44,49-51,54,60-62,65,68]. In three studies a significant improvement in diet quality was achieved [1,61,68]; six studies reported vegetable intake increases [39,44,49-51,54]; in six studies fruit intake increased [39,44,49,50,54,62]; fat intake was reduced in four studies [44,51,60,61]; fewer calories were consumed in one study [60]; frequency of wholegrain product consumption was increased in one study [50]; and consumption of healthy fats increased in one study [65]. Due to the heterogeneity and lack of standard methods to assess dietary intake within the nutrition domain, a meta-analysis was unable to be conducted.

### Weight outcomes

Of the 41 included studies, 12 reported weight-related outcomes (two examined weight exclusively) [37,38,40,47,50,52,54,55,59,64,66,71] and of these 12, four reported significant improvements in these outcomes [38,47,52,66]. The average risk of bias rating for the 12 studies was neutral. A significant reduction in waist-to-hip ratio was reported in one study [38]; in one study BMI decreased significantly [47]; one study reported significant weight loss [52]; and a significant increase in the number of participants trying to lose weight was reported by one study [66]. Due to the variation in aims and measures within the domain of weight, a meta-analysis was not conducted.

## Discussion

The current review identified 41 studies that investigated the impact of lifestyle interventions targeting improvement of health outcomes (specifically physical activity, diet or weight) for students within the tertiary sector. Most studies reported at least one significant improvement in a health outcome variable, with a number of studies having multiple significant impacts. Study results were mostly positive, with at least half of the studies for physical activity and nutrition reporting significant outcomes. These included 18/29 studies examining physical activity that found significant effects including increased physical activity minutes, an increase in the number of days participating in physical activity and also in exercise duration, increased METs and PAR-Q scores and a decrease in barriers to exercise. In addition, results of the meta-analysis suggest the studies targeting moderate physical activity demonstrated significantly greater moderate physical activity levels in the intervention group compared to the control group at post intervention. Of the studies examining nutrition, 50% reported significant improvements, including improved diet quality, increased fruit, vegetable and wholegrain intake, and healthy fats and a reduction in overall fat intake and calories. Of the studies examining body weight, four of 12 resulted in significant outcomes including reductions in weight, BMI, and WHR and/or an increase in the number of participants trying to lose weight.

Interventions spanning a university semester or less (≤12 weeks) generally resulted in a greater number of significant outcomes in comparison to interventions with a duration of more than a semester. In addition, interventions targeting nutrition only resulted in more significant outcomes in comparison to targeting PA, weight or multiple behaviours. For instance, of the ten interventions targeting dietary behaviour, eight (80%) had significant results and the majority of these studies (7/8) were ≤12 weeks. For PA only studies, seven of the 11 (64%) interventions had significant results, and of these seven studies five had a duration of ≤12 weeks. Of the two studies examining weight only interventions, only the 12 week study reported significant results. Furthermore, when targeting a combination of behaviours, 11 of 18 (61%) interventions had significant results, and just over half of these interventions had a duration of ≤12 weeks (6/11).

Less than half of the studies in each category were randomized controlled trials, however there was no trend (based on study counts) towards study design influencing the effectiveness of interventions. The vast majority of the studies were conducted in the USA (n = 31) and therefore the global generalizability of these results must be interpreted with caution.

With few exceptions, participant numbers were surprisingly small given the large institutions from which participants were drawn. Additionally, participants were overwhelmingly female, which may be due in part to the higher percentage of females enrolled in some universities and colleges. This raises questions about the approaches used to recruit participants or the intrinsic appeal of the interventions trialed. Indeed, results from a questionnaire examining gender differences in the health habits of university students showed that males were less interested in nutrition advice and health-enhancing behaviors, suggesting that interventions targeting health behaviors in university/college students may need to be gender-specific to address the different needs and interests of both sexes [15].

The transition from secondary to tertiary education often results in an increase in health risk secondary to a decrease in physical activity and increase in poor dietary choices [74]. For many students, making the transition to tertiary education coincides with more freedom and control over their lives. However, this can contribute to the increases in risk taking behaviors that are evident in this population [75]. With this new-found independence, many students may not have developed skills such as self-efficacy and accountability, leaving them at higher risk of adopting unhealthy behaviors. A number of studies in this review successfully targeted self-efficacy [5,7,39,46,54,57,64] to improve health behaviors.

Interventions that were embedded within university/college courses were effective at improving physical activity, nutrition and weight-related outcomes. Course-embedded interventions involve frequent face-to-face contact with facilitators. It has been suggested that frequent professional contact may improve health outcomes by enhancing vigilance and providing encouragement and support [76]. Additionally, interventions where students received feedback on their progress appeared to be more effective than simply attending lectures or receiving educational resources.

Universities and colleges are an ideal setting for implementation of health promotion programs as they support a large student population at key time for the development of lifestyle skills and behaviors. Students have access to world-class facilities, technology, and highly educated staff including a variety of health disciplines, all of which could contribute to the development of highly effective health promoting interventions. A number of studies in this review utilized university facilities, such as fitness centres and designated walking tracks, showing significant improvements in physical activity outcomes. Besides ease of access for students, use of existing facilities and resources is also cost-effective, which is often a major limitation of health promotion programs.

## Conclusions

This study extends the current literature examining the effectiveness of interventions targeting physical activity, nutrition and weight-loss behaviors amongst university and college students. To the best of the authors’ knowledge it is the first systematic review examining health behaviors of students within a tertiary education setting. Some limitations of the field exist which should be acknowledged. First, the majority of studies examined were conducted in the USA, which may limit interpretations and global generalizability of results. Second, only four of the 41 studies that met the inclusion criteria showed a positive result in meeting the risk of bias validity criteria questions. Also, the potential effect of publication bias must be considered, as the observations made in this review did not include grey literature (e.g., unpublished dissertations).

This review has several strengths. It employed a comprehensive search strategy, adhered to the PRISMA protocol with two reviewers used for the identification and evaluation of studies [30], assessed study risk of bias with two independent reviewers using the Academy of Nutrition and Dietetics Quality Criteria Checklist, and included a meta-analyses for physical activity.

Tertiary education students within the university/college setting are ideal targets for lifestyle interventions aimed at improving health behaviors. Within this setting, students are often surrounded by an abundance of research expertise, multi-disciplinary health professionals, and world-class facilities and resources making this potentially an ideal health-promoting environment. Additionally, students are in a learning environment and are still at an age where health behaviors that impact on health later in life can be improved. Therefore, there is significant scope for implementation of lifestyle interventions to improve the health of this group that represents a significant proportion of our population.

## Additional files

Additional file 1: Table S1. Study Characteristics. Additional file 2: Table S2. Study Results.
