# Supplementary material for: Effectiveness of interventions targeting physical activity, nutrition and healthy weight for university and college students: a systematic review and meta-analysis
Source: Int J Behav Nutr Phys Act. 2015 Apr 1;12:45. doi: 10.1186/s12966-015-0203-7 (PMC4393577; doi:10.1186/s12966-015-0203-7)
Supplement: Additional file 1: Table S1. — Study Characteristics. [file 12966_2015_203_MOESM1_ESM.doc]

# Table S1: Study Characteristics

| **Study / Design / Country / Sample Size** | **Age, y**  **Mean (SD) or range** | **Duration** | **Intervention** | **Retention (%)** | **Risk of Bias Scores +,** ∅ or - |
| --- | --- | --- | --- | --- | --- |
| **Combined** |  |  |  |  |  |
| 1. **Abu-Moghli et al., 2010 / CT / Jordan / n=160** | 21 years | Two 5-day training program | A self-administered, two part questionnaire was conducted to assess the attitude and self-reported behaviors of participants designed to help students make self-directed and autonomous health decisions. | 81.3% | ∅ |
| 1. **Afifi Soweid et al., 2003 / Pre-post / Lebanon / n=32** | 18 – 22 years | 1 semester | A self-administered survey was used to assess attitude and self-reported behavior, on areas such as fitness, nutrition and weight management of students at the beginning and end of the course (3 hours a week for a total of 40 hours a semester). | 53.3% | - |
| 1. **Alpar et al., 2008 / Pre-post / Turkey / n=70** | 17 – 22 years | 2002 - 2006 | A healthy lifestyle behavior survey was conducted during the first week/semester and again during the last week of class in second semester to determine if any improvement was made. The remaining surveys were repeated at the end of every academic year. | 81.4% | ∅ |
| 1. **Bowden et al., 2007 / RCT / USA / n=108** | Diet 1: 19.91  Diet 2: 19.63  Diet 3: 19.93 Diet 4: 20 | 12 weeks | Participants were randomly assigned to 1 of 4 diet groups consisting of Diet 1 (55% carbohydrate, 30% fat, 15% protein), Diet 2 (55% carbohydrate, 30% fat, 15% protein and caloric restriction), Diet 3 (40% carbohydrate, 30% fat, 25% protein), and Diet 4 (40% carbohydrate, 30% fat, 25% protein and caloric restriction) and followed recommended aerobic exercise prescriptions. | 87% | ∅ |
| 1. **Boyle et al., 2011 / CT / USA / n=225** | 21 years | 2007-2008 academic year | The objective of this study was to determine if a course-based, peer education intervention whereby students selected 1 of 2 project options (1 - attempting behavior change with no support (control group), or 2 – improving physical activity levels with professional help) was associated with increases in physical activity and physical fitness. Pre and post assessments and surveys were administered to assess results. | 79% | ∅ |
| 1. **Buscemi et al., 2011 / RCT / USA / n=70** | 19.69 (2.01) | 3 months | Students completed an assessment Weight Related Health Behaviors and were randomized to receive a single 60-min BMI plus a booster phone call or to assessment only. BMI group completed a 50–60-min intervention that aimed to encourage students to increase their physical activity, monitor portion size, increase intake of fruits and vegetables and decrease consumption of fast food and calorie-dense beverages in order to decrease body mass index. | 85.7% | ∅ |
| 1. **Gow et al., 2009 / RCT / USA / n=170** | ≤ 22 years | 6 weeks | Students were randomly assigned to one of four treatment conditions: 1) no treatment, 2) 6-week online intervention 3) 6-week weight and caloric feedback only (via email), and 4) 6-week combined feedback and online intervention. | 94% | + |
| 1. **Hager et al., 2012 / CT / USA / n=2,971** | 19.9 (2.6) | 1.5 years | Participants were required to take an age, health and wellness course either by classroom lecture or online to assess its influence and effectiveness on physical activity and dietary habits/behaviors among students so a comparison of each could be made. | 91% | ∅ |
| 1. **Ince, 2008 / Pre-post / Turkey / n=62** | 22.2 ( 1.6) | 12 weeks | The intervention focused on development of self-regulatory skills, social support, and self-assessment of health-related fitness. The Adolescent Health Promotion Scale and International Physical Activity Questionnaire was administered. | NR | ∅ |
| 1. **Lachausse, 2012 / RCT / USA / n=320** | Online – 26.68  Comparison group – 22.81 | 12 weeks | Online course participants were instructed to visit the My Student Body-Nutrition for at least 2 hours per week over a 12-week period in an effort to determine its effectiveness in decreasing obesity. Participants were then completed the 4 Rate Myself Assessments. | 97.5% | ∅ |
| 1. **LeCheminant et al., 2011 / RCT / USA / n=46** | 18 - 24 years | 1 Academic year | Participants were given a pedometer which was to be worn at all times (besides when sleeping), they were instructed to aim for 10,000 steps daily and had to keep track of their daily step count and number of days not wearing their pedometer. | 67% | ∅ |
| 1. **McClary King et al., 2013 / Pre-post / USA / n=31** | Trainees – 20.2  Interns – 24.2 | 14 weeks | ‘Trainees’ nutrition, Physical Activity behaviors, and perceptions were quantitatively assessed through surveys at pre and post-intervention. Interns’ mentoring perceptions were qualitatively assessed through a focus group interview after the intervention. | 94% | ∅ |
| 1. **Pearman et al., 1997 / CT / USA / n=979** | 27 years | 1 Semester | Through the use of a single survey questionnaire this study assessed whether or not a respondant had attended a college with a compulsory lifetime physical education course (experimental group) or not (control) in order to evaluate participants health knowledge, attitudes and behaviors on their own health status. | 50% | - |
| 1. **Tully & Cupples, 2011 / RCT / Ireland / n=12** | 21.16 (6.17) | 6 weeks | Participants were asked to accumulate 10,000 steps per day and to wear a pedometer every day for 6 weeks. They were to record their daily step count without modifying any aspect of their lifestyle. | 100% | ∅ |
| 1. **Wadsworth et al.,2010 / RCT / USA / n=91** | NR | 6 months | Female participants completed questionnaires measuring Social Cognitive Theory (SCT) variables, and physical activity at baseline, 6 weeks, and 6 months. Through the use of e-mails, a Web site that targeted SCT variables, access to an e-counselor, and access to computer-mediated exercise materials to increase participant knowledge the authors were able to evaluate what effect the SCT web intervention had on female physical activity. | 78% | ∅ |
| 1. **Werch et al., 2007 / RCT / USA / n=155** | 19 (1.12) | Fall 2005 Spring 2006 | Participants were randomly assigned to 1 of 3 interventions: 1) a multiple behavior health contract, 2) one-on-one consultation, or 3) a combination of the 2 in order to have a positive impact on helath behaviors. Data was collected via a computer based questionnaire pre and one month post intervention. | 95% | + |
| 1. **Werch et al., 2008 / RCT / USA / n=303** | 19.2 (1.12) | 3 months | Participants in both groups were first asked to complete the Fitness Behavior Screen. Responses were used to tailor consultation messages to each participant’s specific health habits.  Consultation and Goal Plan participants - were provided with scripted messages by the fitness specialist using a consultation protocol. Consultations lasted approximately 25 min. The consultation was based on the Behavior Image Model. | 95% | ∅ |
| 1. **Yakusheva et al., 2011 / Pre-post / USA / n=1,055** | 18.10 (0.31) | 1 Academic year | Participants conducted surveys (pre and post-intervention) based upon weight and weight management behaviors over their freshman year to determine if their roomates weight and weight management behaviors had a positive or negative impact upon them. | NR | ∅ |
| **Physical Activity** |  |  |  |  |  |
| 1. **Cardinal et al., 2002 / CT / USA / n=540** | LFH; 19.7 (2.8), Prior LFH; 20.4 (2.6), No LFH;21.5(5.7) | 10 weeks | This study involved a 30-hour Lifetime Fitness for Health course which was delivered both in a lecture and lab format to assess students’ leisure-time exercise and advancement through the stages of change for exercise. | 62.8% | ∅ |
| 1. **Cavallo et al., 2012 / RCT / USA / n=134** | < 25 years | 12 weeks | Female participants were randomized into two groups; education-only: participants received access to a physical activity focused website, or the intervention: participants received access to the same website with physical activity self-monitoring as well as enrollment in a Facebook group to determine which group received the most peer social support when trying to increase their physical activity. | NR | ∅ |
| 1. **Claxton et al., 2009 / RCT, USA, n=582** | 19.43 ( 3.41) | 12 weeks | Students were assigned 30 minutes of physical activity homework 3 days a week, or no homework for 12 weeks. Participants completed self-report of physical activity before and at the conclusion of the 12 weeks of physical activity homework. | 63% | ∅ |
| 1. **Fischer et al., 2008 / CT / USA / n=449** | NR | 92 days | The authors examined the effect of certified personal trainer services on exercise behavior by using the trans-theoretical model of behavioral change. Students receiving personal trainer services during the fall semester were cross-matched with students who had not received services. | 63.4% | ∅ |
| 1. **Gieck & Olsen, 2007 / Pre-post / USA / n=41** | 27.36 ( 8.9) | 11 weeks | Participants were required to record daily walking totals and complete 5 bimonthly classes where principles of holistic wellness were discussed. | 36.6% | ∅ |
| 1. **Grim et al., 2011 / CT / USA / n=233** | 21.2 | 10 weeks | Web-based group - received a Social Cognitive Theory behavioral skill-building intervention and exercised 3 days per week in their leisure time. Physical Activity group - received exercise instruction and was required to attend three physical activity labs per week. Comparison group - received health instruction. | 72% | ∅ |
| 1. **Huang et al., 2009 / RCT / Taiwan / n=149** | 18 (.55) | Sept 2004 – April 2005 | Female Freshman were assigned to three groups: an experimental group with stage-matched messages on the website, a generic group with non-stage-matched messages on the website, and a control group that was given only lectures but had no access to the website designed in order to see the effect on the physical activity of young Taiwanese women. | 87% | + |
| 1. **Magoc et al., 2011 / RCT / USA / n=117** | 25 years | 6 weeks | Web-based intervention – Participants received 7 web-based lessons aimed at increasing physical activity. Control group – received minimal physical activity information. Students reported their physical activity levels as well as social cognitive theory constructs at baseline and after study completion. | 89% | + |
| 1. **Martens et al., 2012 / RCT / USA / n=70** | 19.61 (2.41) | 30 minute session | The Brief Motivational Intervention (30-minute, 1-on-1) was delivered in a MI-based framework. Participants received personalized feedback on how their moderate-intensity and vigorous-intensity physical activity compared with national guidelines and perceived barriers to engaging in physical activity. | 98% | ∅ |
| 1. **Sallis et al., 1999 / RCT / USA / n=338** | 24.4 (0.06) | 2 years | The control course was knowledge-oriented. The intervention course taught behavior change skills in weekly lectures and peer-led labs. Physical activity was assessed with 7-Day Physical Activity Recall interviews. | 95% | ∅ |
| 1. **Skar et al., 2011 / RCT / Scotland / n=1,273** | 22.8 (6.7) | 7 months | Action Planning intervention – participants were asked to form up to three plans for PA in the forthcoming term. Coping Plans intervention – participants were asked to anticipate possible difficulties in partaking in PA in advance, plan how to overcome them and to develop up to three plans to deal with these situations. | 45.3% | ∅ |
| **Nutrition** |  |  |  |  |  |
| 1. **Brown et al., 2011 / CT / USA / n=376** | 20 years | 4 months | Participants viewed online vegetable preparation videos and participated in vegetable tasting experiences that featured four target vegetables, one vegetable each month to hopefully increase intake. | 49.5% | ∅ |
| 1. **Chen et al., 1989 / Pre-post / USA / n=272** | 19 - 51 years | 7 weeks | The intervention was a behavioral improvement project which consisted of 8 parts designed to be completed. The overall purpose of the project was to allow students to apply course content to practical and definitive measures of cancer prevention. | NR | - |
| 1. **Evans et al., 2002 / Pre-post / USA / n=5,881** | 14-21 years | 3 years | The primary goal of The Right Bite Nutrition Program was to increase healthful eating behaviors among college students through the use of trained student peer educators and by providing an overall campus approach to a healthful eating environment. | NR | - |
| 1. **Gray et al., 1987/ Pre-post / USA / n=1,207** | 21 | 10 weeks | Cancer course group - Information about pre-course behavior, current cancer experience, and positive behavioral changes was obtained from questionnaires in order to determine what impact these variables may have on the cancer-nutrition link. The same questionnaire was provided to the comparison group who had never taken the cancer course. | NR | - |
| 1. **Ha et al., 2009 / Pre-post / USA / n=80** | 18 – 24 years | Spring semester 2006 | The intervention focused on nutrition knowledge related to prevention of chronic diseases, healthful dietary choices increasing fruit and vegetable consumption, dietary feedback, and interactive hands-on activities. 3-day food records were collected, verified, and analyzed before and after the intervention. | 100% | ∅ |
| 1. **Hekler et al., 2010 / CT / USA / n=100** | “young adults” | January -March 2009 | A Food Frequency Questionnaire was administered at the beginning and end of the four courses. Students in the Food and Society course read selected portions of popular books and essays and watched documentaries highlighting environmental, ethical, social justice, cultural, political, and agricultural issues related to food and food production, and discussed these major themes during class sessions. Students were also required to (1) write an Op-Ed article and (2) create a brief YouTube video focused on themes discussed in the course. | 100% | ∅ |
| 1. **Kolodinsky et al. 2008 / CT / USA / n=16** | 18-20 years | 60 minutes | The experimental design allowed for the measurement of the impact of labelling and labelling plus education, on the food choices of 60 students compared to a randomly selected control group. Of these 16 focus group students, 6 were regularly exposed to nutrition labels; 10 were part of the control group. | 100% | - |
| 1. **Pearce & Cross, 2013 / Pre-post / Australia / n=139** | 18-34 years | 4 weeks | 4^th^ year Pharmacy students received a self-administered questionnaire prior to and after completion of an intensive, 4 week face-to-face nutrition and therapeutic course to determine if it increased their nutritional knowledge and awareness of appropriate every day foods. | 73% | ∅ |
| 1. **Peterson et al., 2010 / CT / USA / n=104** | Pre –  19.58 (1.365)  Post –  19.97 (1.882) | 3 weeks | 21 Healthy choice indicators at point-of-selection were used to increase perceptions of availability of healthy food choices and increase selection of the 10 targeted healthy foods. “The Right Stuff!” logo was on all promotional materials (signs, tray liners, cards) and were placed throughout the University dining hall for maximum exposure. | 38% | ∅ |
| 1. **Reed et al., 2011 / Pre-post / USA / n=278** | “Adults” | 9 days | Pretest fruit and cookie counts were administered 9 days prior to the intervention for each week day lunch and recorded to develop a baseline. Following the initial baseline assessment, a new "point of decision" stimulus (computer video monitor) was added to the counter space between the cookies and fruit. The presentation continued for 9 days, with fruit and cookie choices counted each day. | 100% | ∅ |
| **Weight** |  |  |  |  |  |
| 1. **Harvey-Berino et al., 2012 / CT / USA / n=336** | NR | 12 weeks | The program focused on behavioral strategies to modify eating and exercise behaviors of students interested in losing weight and/or developing a healthy lifestyle. Specific tools included weekly chat meetings with a facilitator, calorie and fat gram recommendations, daily food logs, and exercise guidance. | NR | - |
| 1. **Musgrave & Thornbury, 1976 / Pre-post / USA / n=14** | NR | 2 to 6 months | The objective: that students would be able: a) to select a 1,200-kcal diet from the regular menu served in all food units on the campus and b) to modify their eating patterns following instruction and encouragement in weekly group meetings. The three phases of the program were caloric modification of regular menus, nutrition instruction, and evaluation. | 100% | - |

* Please see Appendix – Table 3: Critical Appraisal Criteria of Study Methodologies for detailed account of Risk of Bias score.
