# Supplementary material for: Effectiveness of interventions targeting physical activity, nutrition and healthy weight for university and college students: a systematic review and meta-analysis
Source: Int J Behav Nutr Phys Act. 2015 Apr 1;12:45. doi: 10.1186/s12966-015-0203-7 (PMC4393577; doi:10.1186/s12966-015-0203-7)
Supplement: Additional file 2: Table S2. — Study Results. [file 12966_2015_203_MOESM2_ESM.doc]

# Table S2: Study Results

Physical Activity

| Study | Sample Size | Outcomes | Results | Significance | p-value |
| --- | --- | --- | --- | --- | --- |
| Abu-Moghli et al., 2010 | n=160 | 1. Physical activity (PA)  a. Score out of eight on self-administered behaviour questionnaire | Mean±SD  (Intervention[I]; Control[C])  Baseline/ Post-intervention  1a. [I] 2.08±0.48/ 2.30±0.59  [C] 2.02±0.64/ 2.02±0.63  *Observed power = 0.05* | No significant difference between [I] and [C] at the completion of the study for physical activity. | 1.a. p=0.99 |
| Afifi Soweid et al., 2003 | n=32 | 1. Fitness | Mean  Pre-test/Post-test  1. 2.82/3.14  *Difference = 0.32*  *% change pre to post = 11.3%* | No significance reported | 1. NR |
| Alpar et al., 2008* | n=70 | 1. Health promotion lifestyle profile’ (HPLP) developed by Pender et al. (1987).  a. Exercise | Mean (SD)  Entrance to school/Graduation  1a. 10.40 (2.37)/ 12.14 (2.42) | Significant differences to total HPLP and subscale scores (exercise) between when they entering and finishing the nursing program. | 1.a. p=0.000 |
| Bowden et al., 2007 | n=108 | 1. Physical activity  a. Comparison of average minutes of physical activity per week between four intervention groups | Mean±SD  (Intervention 1[I1]; Intervention 2[I2];  Intervention 3[I3]; Intervention 4[I4])  Baseline/Post-intervention (Average minutes/week)  1a. [I1] 129.87±106.13; [I2] 133.37±78.23;  [I3] 133.44±94.34; [I4] 122.70±74.64 | No significant difference between [I] groups for physical activity (p=0.632). | 1.a. p=0.632 |
| Boyle et al., 2011* | 1. n=225 | 1. 1.Total Physical Activity 2. 2. Energy Expenditure 3. 3. VO2 max 4. 4. Flexibility 5. 5. RMCP (lbs) 6. 6. RMLP (lbs)   RMCP = Rep Max Chest Press  RMLP = Rep Max Leg Press | Mean(SD)  (Control[C]; Treatment [T])  Pre-test/Post-test  Women –  1. [C] 251.19(3.09)/147.91(2.40);  [T] 199.53(2.75)/194.98(2.34)  2. [C] 2.69(3.24)/1.55(2.45); [T] 2.29(3.02)/2.04(2.51)  3. [C] 36.69(5.03)/37.14(4.45); [T] 37.33(5.20)/38.08(4.81)  4. [C] 12.92(3.53)/1308(2.96); [T] 12.64(3.04)/13.73(2.22)  5. [C] 66.65(17.00)/82.19(31.07);  [T]73.52(26.73)/81.81(19.92)  6. [C] 216.03(62.42)/243.51(67.11);  [T] 233.74(73.30)/260.89(77.37)  Men –  1. [C] 208.93(2.40)/181.97(2.95);  [T] 169.82(3.09)/223.87(2.51)  2. [C] 2.57(2.45)/2.09(2.95); [T] 2.00(3.24)/2.69(2.57)  3. [C] 41.50(4.68)/42.25(4.65); [T] 41.38(6.29)/44.13(8.44)  4. [C] 11.73(1.71)/11.51(2.50 ; [T] 10.94(2.60)/12.09(2.52)  5. [C] 192.31(52.07)/224.27(74.97);  [T] 210.65(73.71)/231.05(69.77)  6. [C] 367.39(99.53)/389.13(125.06);  [T] 453.50(97.26)/487.51(88.13) | Women in the treatment group had significantly smaller PA reduction | 1. p<0.05 |
| Buscemi et al., 2012 | n=70 | 1. Vigorous physical activity  2. Moderate physical activity | Mean(SD)  Baseline BMI / Follow-up BMI  *d*  Group 1 –  1. 75.88(119.28)/68.97(97.71) *0.07*  2. 90.21(147.27)/69.62(79.11) *0.17*  Group 2 –  1. 70.88(127.12)/74.87(194.50) *-0.02*  2. 114.14(159.46)/66.0(93.67) *0.37* | No significance. | 1. p>0.05  2. p>0.05 |
| Cardinal et al., 2002* | n=540 | 1. Pre-contemplation  2. Contemplation  3. Preparation  4. Action  5. Maintenance  Lifetime Fitness for Health (LFH) | Mean±SD  (LFH[L]; Prior LFH[P]; No LFH[N])  Baseline/Post-intervention  1. [L] 11.9±17.2/21.5±21.4; [P] 31.5±0/19.0±0;  [N] 7.0±2.8/14.5±13.4.  2. [L] 26.3±16.3/39.0±25.8; [P] 31.6±0/19.0±0;  [N] 9.0±5.7/14.0±1.4.  3. [L] 22.8±14.6/35.9±23.5; [P] 27.0±17.2/43.2±44.6;  [N] 23.0±14.4/42.9±27.2.  4. [L] 44.4±19.3/51.2±19.9; [P] 39.1±16.5/41.6±19.2;  [N] 51.3±23.6/42.1±23.4  5. [L] 54.5±23.2/58.9±27.2; [P] 58.8±28.3/55.6±21.7;  [N] 48.4±23.2/51.4±24.2 | Exercise METS showed significant change for 16 of the 20 contrast measured.  Significant two-way interaction between stage and time.  Significant differences across groups for stage of change behaviour from pre- to post-intervention | p<0.05  p<0.001  p<0.05 |
| Cavallo et al., 2012* | n=134 | 1. Physical Activity  a. Total kcal  b. Heavy kcal  c. Moderate kcal  d. Light kcal | Mean(SD)  (Intervention[I]; Control[C])  Baseline/Post-test survey  *Group time interaction effect/Time effect*  1.a. [C]: 1706.23(1315.44)/2248.98(1541.19)  [I]: 1646.39(973.68)/2394.75(1448.00)  *1.57/12.13*  b. [C]: 155.97(347.70)/378.23(731.36)  [I]: 151.79(333.57)/298.21(575.32)  *0.35/9.19*  c. [C]: 166.69(343.15)/272.78(604.52)  [I]: 81.03(215.81)/253.79(646.08)  *0.26/6.80*  d. [C]: 25.89(72.60)/61.69(134.95)  [I]: 76.79(177.79)/81.25(182.55)  *0.69/2.54* | NR | Group time interaction effect/Time effect  1.a. p=0.52/  p<0.000  b. p=0.55/  p=0.003  c. p=0.61/ p=0.01  d. p=0.41/ p=0.11 |
| Claxton & Wells, 2009 | n=582 | 1. Moderate Activity  2. Vigorous Activity  3. Muscular Strength/Endurance Activity  4. Flexibility Activity  5. Weight Management Activity | Mean(SD)  (PA Homework[I]; No PA Homework [C])  Pre-test/Post-test  *Change Mean (SD) – [I];[C]*  1. [I]: 2.31(1.75)/3.11(1.67); [C]: 2.36(1.82)/2.82(1.60)  *0.73(1.88); 0.47(1.85)*  2. [I]: 1.79(1.86)/2.19(1.73); [C]: 1.95(2.02)/2.19(1.82)  *0.42(1.60); 0.23(1.82)*  3. [I]: 1.68(1.92)/2.10(1.90); [C]: 1.82(1.88)/1.97(1.84)  *0.46(1.80); 0.15(1.86)*  4. [I]: 1.54(1.94)/1.78(1.93); [C]: 1.70(2.82)/1.90(1.89)  *0.28(2.03); 0.19(1.87)*  5. [I]: 1.69(1.98)/2.17(2.10); [C]: 2.20(2.25)/2.19(1.98)  *0.53(2.28); 0.07(2.28)* | Number of days in which the PA homework group engaged in physical activity to manage or lose weight | 5. p=.0293 |
| Fischer & Bryant, 2008* | n=449 | 1. Cognitive POC  2. Behavioral POC  3. Decisional Balance  4. Coping self- efficacy (SE)  5. Scheduling SE  6. Task SE | Mean(SD)  Experimental Group[E]; Control Group[C]  T1/T2 *Effect Size*  1. [E]: 69.42(9.23)/67.61(8.89) *0.20*  [C]: 65.71(12.86)/59.16(13.70) *0.53*  2. [E]: 71.00(8.96)/67.45(8.23) *0.43*  [C]: 70.55(12.51)/63.42(12.20) *0.81*  3. [E]: 1.84(0.94)/1.71(0.90) *0.13*  [C]: 2.27(1.04)/1.77(0.90) *0.60*  4. [E]: 6.74(1.92)/6.35(1.89) *0.21*  [C]: 6.88(1.62)/6.30(2.16) *0.35*  5. [E]: 7.01(2.14)/6.49(2.23) *0.27*  [C]: 7.25(1.86)/6.10(2.59) *0.57*  6. [E]: 8.25(1.12)/8.08(1.52) *0.14*  [C]: 8.40(1.18)/8.35(1.24) *0.03* | Control group demonstrated a significant regression in state of exercise change scores & experimental did not | Experimental group:  1. p=0.272  2. p=0.25  3. p=0.461  4. p=0.252  5. p=0.145  6. p= 0.454  Control group:  1. p=0.006  2. p=0.000  3. p=0.002  4. p=0.059  5. p=0.004  6. p=0.863 |
| Gieck & Olsen, 2007 | n=41 | Physical wellness | Mean(SD)  (Knowledge[K]; Self-efficacy[SE])  Pre-test/Post-test  [K]: 51.17(5.9)/73.29(2.2); [SE]: 35.47(9.84)/45.12(3.2) | No significant group differences | 1. p=.07 |
| Gow et al., 2010 | n=170 | 1. Questionnaires  a. International physical activity questionnaire  i. total  ii. walk  iii. moderate  iv. vigorous | Mean±SD  (Intervention 1[I1]; Intervention 2[I2]; Intervention 3[I3]; Control[C])  Baseline/Post  2.a.i [I1] 2782±2845/4119±563; [I2] 4725±56/3059±541; [I3] 4678±6041/5005±576; [C] 3289±3108/ 4304±560  2.a.ii [I1] 937±675/2203±466; [I2] 2278±3414/1377±446; [I3] 2258±3371/2588±475; [C] 1382±1718/ 2145±461  2.a.iii [I1] 620±1812/914±233; [I2] 828±1315/ 899.30±227; [I3] 629±1302/1128±233;  [C] 505±1033/772±230  2.a.iv [I1] 1037±1468/939±229; [I2] 2786±4407/909±223; [I3] 1942±3236/1479±229; [C] 1138±1305/ 1270±226 | NR | NR |
| Grim, Hortz & Petosa, 2011* | n=233 | 1. Vigorous physical activity days  2. Moderate physical activity days  3. Self-regulation  4. Friend social support  5. Self-efficacy  6. Outcome expectancy value | Mean(SD)  (Web[W]; Physical Activity [PA]; Health[H])  Pre-test /Post-test  1. [W]: 1.39(1.79)/2.21(1.58);  [PA]: 1.39(2.03)/2.36(1.84); [H]: 0.59(1.17)/0.39(0.61)  2. [W]: 2.77(2.08)/3.84(3.89);  [PA]:4.65(2.26)/3.53(2.03); [H]:2.77(4.09)/2.28(2.19)  3. [W]: 121.6(29.6)/171.5(26.0);  [PA]: 118.6(24.5)/155.6(25.5);  [H]:105.0(37.7)/123.2(39.9)  4. [W]: 28.7(10.0)/32.2(11.4);  [PA]: 29.7(9.2)/31.1(10.0); [H]: 26.4(9.8)/27.9(11.2)  5. [W]: 918.7(244.5)/968.8(210.4);  [PA]: 982.7(231.9)/982.4(243.5); [H]: 827.9(305.0)  6. [W]: 699.9(223.9)/722.8(221.4)  [PA]:725.4(207.0)/749.7(219.2);  [H]: 605.8(193.1)/643.7(225.4) | Outcomes 1 (VigPA), 3 (SR) and 6 (OExV) all changed significantly in the web-based and PA course groups. | 1. p<.01  3. p<.01  6. p<.01 |
| Hager et al., 2012* | n=2971 | 1. Physical activity outcomes (self-report)  a. Number of days performing moderate physical activity  (scale of 1-7)  b. Number of minutes per day performing moderate physical activity (Scale of 1-4; 10, 20, 30 >30 mins)  c. Number of days performing vigorous physical activity  (scale of 1-7)  d. Number of minutes per week performing vigorous physical activity (Scale of 1-4; 10, 20, 30 >30 mins)  e. Overall activity  (scale of 1-10)  f. Estimated VO_2_ max | Mean±SD  (Intervention 1[I1] lecture; Intervention 2[I2] online)  *Change (%) – [I1]; [I2]*  1a. [I1] 4.8±1.5/ 5.1±1.3; [I2] 5.0±1.5/ 5.1±1.4 *0.3; 2*  1b. [I1] 2.6±1.0/ 2.8±1.0; [I2] 2.6±1.0/ 2.8±1.0 *7.7; 7.7*  1c. [I1] 3.0±1.5/ 3.1±1.3; [I2] 3.2±1.5/ 3.1±1.5 *3.3;-3.1*  1d. [I1] 3.0±1.0/ 2.9±1.0; [I2] 3.1±1.0/ 3.0±1.0 *-3.3; -3.2*  1e. [I1] 5.1±2.2/ 5.8±1.9; [I2] 5.1±2.3/ 5.6±2.0 *13.7; 9.8*  1f. [I1] 46.9±7.2/ 48.0±6.7; [I2] 46.1±7.3/ 47.0±6.9 *2.3; 2.0* | Significant increase in overall PA, daily minutes of moderate PA and number of days participating in days participating in at least 10 minutes of moderate PA. VO_2_max also increased significantly. | 1.a. p<0.001  1.c. p=0.001  1.e. p<0.001  1.f. p=0.002 |
| Huang et al., 2009* | n=149 | 1. Physical Activity  2. Knowledge of fitness  3. Benefit of exercise  4. Barriers to exercise  5. Self-efficacy | Mean(SD)  Stage-matched[S]; Generic[G]; Control[C]  Pre-test/Post-test/Delayed Post-test  1. [S] 578.27(562.42)/540.06(826.83)/ 920.10(1092.88)  [G] 840.96(745.95)/ 634.84(575.87)/ 442.72(491.16  [C] 1274.26(850.21)/1172.33(661.62)/669.17(672.46)  2. [S] 12.90(1.33)/12.83(1.63)/13.02(1.45)  [G] 12.94(1.49)/13.23(1.48)/13.09(1.55)  [C] 13.00(1.41)/13.11(1.72)/12.26(2.02)  3. [S] 42.36(5.47)/40.96(6.85)/41.43(5.88)  [G] 42.10(6.95)/ 40.22(8.19)/ 40.47(5.75)  [C] 42.04(6.99)/41.48(7.06)/41.36(5.52)  4. [S] 47.45(9.71)/46.85(11.09)/ 45.02(9.85)  [G] 47.82(8.57)/ 45.06(10.00)/ 46.53(10.74)  [C] 48.00(9.33)/ 46.72(9.90)/ 50.48(10.28)  5. [S] 41.76(12.94)/42.70(11.29)/43.02(12.35)  [G] 42.04(13.71)/ 40.43(11.31)/40.55(11.74)/  [C] 46.47(12.27)/45.46(9.39)/41.89(11.85) | Significantly higher level of exercise SE in stage matched group compared with other two | 5. p=0.002 |
| Ince et al., 2008* | n=62 | 1. Health promoting behaviour (self-report; completion of adolescent health promotion scale)  a. Exercise behaviour  2. Physical activity outcome (MET-MIN/wk)  a. Walking  b. Moderate physical activity  c. Vigorous physical activity  d. Total physical activity | Mean±SD  Baseline/ Post-intervention  1a. 10.5±4.1/ 14.1±2.8  Mean[Median]  Baseline/ Post-intervention  2a. 929.2[693.0]/ 996.1[693.0]  2b. 250.9[120.0]/ 318.6[240.0]  2c. 521.4[0.0]/ 1393.5[960.0]  2d. 1701.5[1447.5]/ 2708.2[2026.0] | Significant changes from baseline to post-intervention for all outcomes except walking. | 1.a. p=0.001  2.b. p=0.01  2.c. p=0.001  2.d. p=0.001 |
| Lachausse, 2012* | n=320 | 1. Aerobic exercise  a. Change in frequency/week of aerobic exercise  2. Attitude toward exercise  a. Change in attitude toward exercise  3. Self-efficacy  a. Self-efficacy to perform exercise | Mean±SD  (Intervention 1[I1] online course; Intervention 2[I2] on-campus course; Comparison group[C])  Baseline/ Post-intervention  1a. [I1] 3.17±1.55/ 3.48±1.73; [I2] 3.46±1.77/ 3.49±1.72;  [C] 3.42±1.79/ 3.35±1.76  2a. [I1] 5.87±0.88/ 6.10±0.77; [I2] 5.98±0.89/ 6.04±0.84;  [C] 5.95±0.89/ 5.90±0.89  3a. [I1] 46.74±20.18/ 48.57±21.56  [I2] 46.1±20.66/ 42.98±19.36;  [C] 44.63±19.36/ 46.54±19.36 | [I1] significantly increased exercise overtime but [I2] and [C] did not.  There were no other significant differences. | 1.a. p=0.001  p>0.05 |
| LeCheminant et al., 2011 | n=46 | 1. VO2max(ml.kg-1.min-1)  2. Treadmill Time (min)  3. RPEmax  4. Heart Rate max (bpm) | Mean±SD  (Control[C]; Pedometer [P])  September/April/change  Change±SD  1. [C]: 40.7±9.0/ 40.3±8.4/ -0.9±4.0  -0.9±4.0 0.17±3.8  2. [C]: 11.1±3.2/ 10.7±2.7/ -0.5±1.5 -0.5±1.5  [P]: 12.9±2.2/ 12.5±2.4/ -0.3±1.2 -0.3±1.2  3. [C]: 18.9±1.2/ 18±1.8/ -0.8±1.3 -0.8±1.3  [P]: 18.2±1.4/ 17.7±2.2/ -0.4±1.7 -0.4±1.7  4. [C]: 191.9±8.2/ 187.9±13.2/ -4.5±13.11  -4.5±13.11  [P]: 195.3±9.8/ 193.3±8.6/ -2.0±5.2 -2.0±5.2 | No significance | 1. p>.05  2. p>.05  3. p>.05  4. p>.05 |
| Magoc et al., 2011* | n=117 | 1. Physical Activity  a. Moderate Days  b. Moderate Minutes  c. Vigorous Days  d. Vigorous Minutes  2. SCT Constructs  a. Self-efficacy  b. Goals  c. Plans  d. Expectancies  e. Family Social Support  f. Friends’ Social Support | Mean(SD)  Pretest/Posttest  1a. 1.57 (1.82) 2.59 (2.14)  1b. 4.87 (2.96) 6.00 (2.60)  1c. 1.80 (1.66) 2.15 (1.61)  1d. 5.62 (3.64) 5.75 (2.77)  2a. 18.22 (3.94) 17.70 (4.20)  2b. 15.04 (5.02) 16.14 (5.45)  2c. 12.45 (3.82) 13.17 (3.88)  2d. 48.07 (15.80) 53.23 (14.95)  2e. 10.68 (5.47) 11.60 (5.79)  2f. 12.42 (5.61) 12.80 (5.90) | Vigorous days in treatment group by time interaction | 1.c. p <.001 |
| Martens et al., 2012* | n=70 | 1. Days Per Week of Vigorous Physical Activity  2. Days Per Week of Moderate Physical Activity  3. Minutes Per Week of Vigorous Physical Activity  4. Minutes Per Week of Moderate Physical Activity | Mean  (Motivational Interviewing[MI]; Control[C])  Baseline/Follow-Up  1. [M]: 0.5/1.75; [C]: 0.1/0.75  2. [M]: 2.5/2.75; [C]: 1.1/1.9  3. [M]: 19/65; [C]: 10/19  4. [M]: 50/80; [C]: 22/40 | Statistically significant differences between mean vigorous (1) and mean moderate (2) PA days/week at baseline (MI group higher) | 1. p<.05  2. p<.05 |
| McClary King et al., 2013* | n=31 | 1. Exercise barriers  2. Sedentary behaviour  3. Perceived health benefits | Mean  (On-campus[ON]; Off-campus[OFF]; Combined[C])  Pre-intervention/Post-intervention  1. [ON]: 22.75/19.50; [OFF]: NR/23.39  2. [C]: 184.79/326.86  3. [C]: 31.57/66.31 | 1. Significant interaction effects  of the intervention and residency.  1. Post-intervention, trainees living [ON] perceived significantly fewer exercise barriers compared with trainees living [OFF] | 1. p<0.01  1[ON]. p<0.05 |
| Pearman et al., 1997 | n=979 | 1. Physical activity behaviours (mean score out of 5; 1=rarely or never, 5=more than once/week)  a. Active sports  b. Physical exercise  c. Jogging/running  d. Swimming/walks  e. Garden, fish, hunt | Mean  Intervention[I] College A (health course); Control [C] College B (no health course)  Post-intervention  1.a [I] 2.9; [C] 2.9  1.b [I] 4.1; [C] 4.0  1.c [I] 2.9; [C] 2.5  1.d [I] 3.5; [C] 3.4  1.e [I] 2.2; [C] 2.4 | Significant difference between groups for jogging/running. [I] more likely to jog/run than [C].  Significant difference between groups for gardening, fishing, and hunting. [C] more likely garden, fish or hunt than [I].  Men were more likely to participate in active sports and running and women more likely to participate in swimming or take long walks (p<0.01). | 1.c. p<0.01  1.e. p<0.01  Men: p<0.01  Women: p<0.01 |
| Sallis et al., 1999* | n=338 | 1. Total physical activity in leisure time (kcal.kg.week)  2. Vigorous activity in leisure time (hour/week)  3. Moderate activity in leisure time (hour/week)  4. Strengthening exercise (min/week)  5. Flexibility (min/week) | Mean(SD)  Men[M]; Women[W]  Condition x time/Activity status x time/Condition x activity status x time  1. [M]: 0.03(0.87)/0.03(.58)/0.90(.34)  [W]: 4.59(.03)/0.85(.36)/3.76(.05)  2. [M]: 0.03(.87)/0.03(.87)/1.14(.29)  [W]: 1.87(.17)/0.98(.32)/0.34(.56)  3. [M]: 0.86(.36)/0.01(.92)/0.47(.50)  [W]: 1.59(.21)/0.53(.47)/0.17(.68)  4. [M]: 1.10(.32)/8.95(.001)/0.30(.58)  [W]: 26.03(.001)/2.90(.09)/3.28(.07)  5. [M]: 0.72(.40)/0.02(.90)/.65(.42)  [W]: 11.31(.001)/0.57(.45)/0.02(.88) | No significant effects on men; among women, the intervention increased total physical activity during leisure, strengthening exercises, and flexibility exercises. | Men:  1. p=0.87  2. p=0.87  3. p=0.36  4. p=0.32  5. p=0.40  Women:  1. p=0.03  2. p=0.17  3. p=0.21  4. p=0.001  5. p=0.001 |
| Skar et al., 2011* | n=1273 | 1. Physical Activity  2. Intention  3. Perceived Behavioural Control | Mean(SD)  Timepoint 1/Timepoint 2  1. 2.95(2.37)/2.92(2.24)  2. 5.71(1.26)/4.47(0.83)  3. 5.45(1.33)/5.51(1.36) | Intention at Time point 2 | 2. p=0.02 |
| Tully & Cupples, 2011* | n=12 | 1. Number of steps per day  2. Predicted VO2max (ml/kgmin) | Mean(SD)  10,000 step group[SG]; Control group[CG]  Pre-intervention/Post-intervention  1. [SG]: 8824.1(5379.3)/12635.9(6851.3)  [CG]: 7263.8(2837.5)/7656.6(3931.5)  2. [SG]: 34.14(4.62)/31.77(6.34)  [CG]: 32.05(4.59)/33.33(4.41) | 10,000 step group taking significantly more steps after 6 weeks | 1. p=0.03 |
| Wadsworth et al., 2010* | n=91 | 1. Frequency of physical activity (days/week)  2. Physical activity self-efficacy – 15-item self-report questionnaire (perceived confidence to overcome barriers to physical activity) | Mean±SD  Intervention [I]; Control [C]  Baseline/ 6 weeks/ 6 months  1. [I] 2.31±2.03/ 3.13±2.18/ 3.06±2.22  [C] 1.50±1.42/ 1.92±1.84/ 2.03±1.83  2. [I] 59.47±15.86/ 54.31±17.45/ 61.10±21.09  [C] 55.02±17.45/ 49.77±17.87/ 58.28±16.26 | Significantly greater frequency of physical activity in [I] than [C] at 6 weeks but not 6 months. | 1. p=0.026 |
| Werch et al., 2007* | n=155 | 1. Physical activity and exercise  a. Length of exercise  b. 30-day vigorous physical activity  c. 30-day moderate physical activity  d. 7-day average strenuous exercise  e. 7-day average moderate exercise | Mean±SE  Intervention 1[I1] Contract group; Intervention 2[I2] Consult group; Intervention 3[I3] Contract + Consult group  Baseline/ Post-intervention  1.a [I1] 4.14±0.22/ 4.18±0.20; [I2] 3.98±0.23/ 4.19±0.20;  [I3] 3.55±0.22/ 3.84±0.20  1.b [I1] 4.70±0.21/ 4.58±0.21; [I2] 4.15±0.21/ 4.08±0.22;  [I3] 4.14±0.21/ 4.37±0.21  1.c [I1] 4.84±0.26/ 4.94±0.20; [I2] 4.08±0.26/ 5.06±0.21;  [I3] 4.33±0.26/ 5.06±0.21  1.d [I1] 4.04±0.29/ 4.14±0.25; [I2] 3.48±0.30/ 3.50±0.26;  [I3] 3.84±0.29/ 4.00±0.25  1.e [I1] 4.84±0.37/ 5.14±0.32; [I2] 3.81±0.38/ 4.83±0.32;  [I3] 4.18±0.37/ 5.37±0.32 | All three groups showed a significant increase in length of exercise, 30-day moderate physical activity, 7-day average moderate physical activity | 1.a. p=0.02  1.c. p=0.00  1.e. p=0.00 |
| Werch et al., 2008 | n=303 | 1. Physical activity and exercise  a. Length of exercise  b. 30-day vigorous physical activity  c. 30-day moderate physical activity  d. 7-day average strenuous exercise  e. 7-day average moderate exercise | Mean±SE  Intervention [I]; Control [C]  Baseline/ Post-intervention  1.a [I] 3.46±0.15/ 3.66±0.13; [C] 3.67±0.15/ 3.75±0.13  1.b [I] 3.71±0.15/ 3.97±0.14; [C] 3.96±0.15/ 4.19±0.14  1.c [I] 4.32±0.15/ 4.52±0.15; [C] 4.72±0.15/ 4.46±0.14  1.d [I] 3.44±0.19/ 3.56±0.18; [C] 3.80±0.19/ 3.83±0.17  1.e [I] 5.18±0.22/ 5.25±0.19; [C] 5.15±0.22/ 5.22±0.19 | No significant results. | 1.a. p>0.05  1.b. p>0.05  1.c. p>0.05  1.d. p>0.05  1.e. p>0.05 |
| Yakusheva et al., 2011* | n=1055 | 1.Used the gym  2. Exercised outside | Mean(SD)  Baseline/follow up  1. 2.20(2.02)/2.55(1.93)  2. 2.55(2.38)/1.15(1.56) | Exercised outside at baseline. | 2. p=.003 |

Nutrition

| Study | Sample Size | Outcomes | Results | Significance | p-value |
| --- | --- | --- | --- | --- | --- |
| Abu-Moghli et al., 2010* | n=160 | 1. Type of diet  a. Score out of nine on self-administered behaviour questionnaire  2. Nutritional habits  a. Score out of eight on self-administered behaviour questionnaire | Mean±SD  (Intervention[I];Control[C])  Baseline/ Post-intervention  1a. [I] 2.51±0.43/ 2.82±0.46; [C] 2.65±0.42/ 2.65±0.42  2a. [I] 2.63±0.44/ 2.81±0.43; [C] 2.57±0.49/ 2.57±0.49 | Significant difference between [I] and [C] at the completion of the study for type of diet and nutritional habits | 1. p=0.00  2. p=0.00 |
| Afifi Soweid et al., 2003 | n=32 | 1. Nutrition and weight management | Mean  Pre (A)/Post (B)   1. 2.59/2.93 | No significance reported | NR |
| Alpar et al., 2008 | n=70 | 1. Health promotion lifestyle profile’ (HPLP) developed by Pender et al. (1987).  b. Nutrition | Mean (SD)  Entrance to school/Graduation  1b. 6.03 (1.71)/ 6.31 (1.40) | No significance | 1.b.p=0.262 |
| Buscemi et al., 2012 | n=70 | 1. Fruit intake  2. Vegetable intake  3. Sweet food intake  4. Fast food intake  5. Sugar-sweetened beverage | Mean(SD)  Baseline BMI / Follow-up BMI  Group 1[G1]; Group 2[G2]  1. [G1]: 1.96(2.60)/2.39(1.91); [G2]: 1.81(1.75)/1.93(1.46)  2. [G1]: 1.79(2.63)/2.93(2.45); [G2]: 2.18(1.65)/2.27(2.13)  3. [G1]: 2.38(0.43)/2.06(0.51); [G2]: 2.40(0.50)/2.27(0.667)  4. [G1]: 2.13(0.47)/1.88(0.44); [G2]: 2.10(0.43)/2.02(0.53)  5. [G1]: 2.23(0.53)/2.07(0.54); [G2]: 2.25(0.42)/2.30(0.54) | No significance | 1. p>0.05  2. p>0.05  3. p>0.05  4. p>0.05  5. p>0.05 |
| Bowden et al., 2007 | n=108 | 3. Dietary intake  a. Comparison of average daily carbohydrate (%) intake between four intervention groups  b. Comparison of average daily protein (%) intake between four intervention groups  c. Comparison of average daily fat (%) intake between four intervention groups  d. Comparison of average daily total energy (kCal) intake between four intervention groups | Mean±SD  (Intervention 1[I1]; Intervention 2[I2]; Intervention 3[I3]; Intervention 4[I4])  Baseline/ Post-intervention  3a. [I1] 51.12±5.19; [I2] 53.92±6.33;  [I3] 41.21±7.38; [I4] 42.73±6.31  3b. [I1] 16.03±2.82; [I2] 15.62±2.33;  [I3] 25.06±5.91; [I4] 26.64±5.35  3c. [I1] 32.72±5.28; [I2] 30.54±6.31;  [I3] 33.71±6.75; [I4] 30.64±5.30  3d. [I1] 1621±492; [I2] 1465±599;  [I3] 1222±442; [I4] 1487±557 | No significant difference in energy intake between groups. No other significances were reported for dietary outcomes. | 1.d. p=0.384 |
| Brown et al., 2011* | n=376 | 1. Vegetable intake  a. Change in total and target vegetable intake from baseline to post-intervention  b. Change individual vegetable intake from baseline to post-intervention  2. Readiness to change vegetable intake  a. Change in stage (pre-contemplation, contemplation, preparation, action, maintenance) from baseline to post-intervention  3. Knowledge and self-efficacy of vegetable preparation  a. Change in knowledge of vegetable preparation from baseline to post-intervention  b. Change in self-efficacy of vegetable preparation from baseline to post-intervention | Mean±SD  Baseline/ Post-intervention  1a. Total vegetables: 1.63±1.24/ 1.63±1.3  1b. Onions, potatoes, salad greens: No change  Asparagus: 0.02±0.36/ 0.03±0.19  % of subjects consuming asparagus <once per month: 68.3%/ 57.5%  2a. The mean stage of change score increased from 2.5±1.34 to 2.7±1.27  % of subjects in pre-contemplation stage decreased from 32.8% to 21.5%  % of subjects in action group increased from 2.7% to 7.5%  Contemplation and preparation stages also showed increases but maintenance did not.  3a. Knowledge of vegetable preparation increased from 59.2% to 73.1%  3b. Self-efficacy of vegetable preparation at the end of the intervention was associated with both total and target vegetable intake. | Significant association for both total (i) and target (ii) vegetable intake.  Significant change in asparagus consumption (i) and % of subjects consuming asparagus < once per month. No other changes in individual vegetable consumption were significant.  Significant change in mean stage of change score. Other significances NR.  Significant increase the % of subjects reporting knowledge of vegetable preparation | 1.a(i). p=0.002  1.a(ii). p=0.024  1.b(i). p=0.016  1.b(ii). p<0.0001  2.a. p<0.019  3.a. p<0.001 |
| Chen et al., 1989 | n=272 | 1. Dietary habits – score of 1-100  a. Success score for general dietary habits (other than caffeine)  b. Success score for general dietary habits (other than fibre)  c. Success score for reduction or avoidance of caffeine  d. Success score for increased fibre consumption | Mean success score (out of 100) for achieving their behavioural risk reduction strategies  1a. 73.3  1b. 71.6  1c. 79.1  1d. 76.3 | No change data reported to determine effect of intervention. | NR. |
| Evans et al., 2002* | n=5881 | 1. Eating behaviours  a. Fruit and fruit juice consumption (self-report)  b. Vegetable consumption (self-report)  c. Fat intake (self-report) | Data NR | Significant increase between baseline and post intervention in fruit and fruit juice consumption and vegetable consumption  Significant decrease between baseline and post intervention in fat intake | 1.a. p<0.0001  1.b. p<0.05  1.c. p<0.0001 |
| Gow et al., 2010* | n=170 | 1. Questionnaires  a. Block food screener  i. fat  ii. fibre  iii. fruit/veg  b. Eating behaviour questionnaire (cigs/day) | Mean±SD  (Intervention 1[I1]; Intervention 2[I2]; Intervention 3[I3]; Control[C])  1a (i). [I1] 105.95±18.04/104.60±2.30;  [I2] 104.70±28.59/ 102.58±2.19;  [I3] 106.30±22.25/102.03±2.31;  [C] 95.39±19.16/104.56±2.30  1a (ii). [I1] 14.44±4.70/15.81±0.57;  [I2] 15.48±4.32/ 15.66±0.56;  [I3] 14.54±5.00/15.09±0.58;  [C] 15.35±4.23/14.80±0.57  1a(iii). [I1] 1.32±1.67/1.73±0.21;  [I2] 1.87±1.54/1.63±0.21;  [I3] 1.46±1.64/1.57±0.22;  [C] 1.80±1.43/1.44±0.21  1b. [I1] 0.67±2.15/2.36±0.61;  [I2] 1.58±4.04/2.36±0.41;  [I3] 1.48±3.23/3.76±0.44a;  [C] 1.27±4.27/ 1.45±0.56a | Significant difference in post scores between [I3] and [C] for eating behaviour | 1.b. p<0.05 |
| Gray et al., 1987 | n=1207 | 1. Dietary intake  a. % of subjects who reduced fat intake during intervention  b. % of subjects who increased fibre intake during intervention | % of respondents  (Intervention [I]/ Control [C])  1a. Female: [I] 47%; [C] 25%  Male: [I] 49%; [C] 14%  1b. Female: [I] 48%; [C] 12%  Male: [I] 35%; [C] 9% | NR | NR |
| Ha et al., 2009* | n=80 | 1. Dietary intake  a. Change in vegetable intake  i. Total  ii. Fresh  iii. Starchy  iv. French fries  v. Vegetable juice  b. Change in fruit intake  i. Total  ii. Fresh  iii. Canned  iv. Fruit juice  c. % of subjects consuming <1 cup of total or fresh vegetables  d. % of subjects consuming <2 cups total fruit or <1 cup of fresh fruit | Mean±SD  Baseline/ Post-intervention  1a. i. 0.77±0.62/ 1.52±1.03  ii. 0.46±0.50/ 1.2±0.93  iii. 0.30±0.33/ 0.29±0.40  iv. 0.15±0.28/ 0.07±0.15  v. 0.01±0.07/ 0.02±0.15  1b. i. 0.94±0.92/ 1.33±0.99  ii. 0.43±0.61/ 0.99±0.85  iii. 0.06±0.15/ 0.05±0.15  iv. 0.45±0.64/ 0.32±0.47  1c. Total: 72%/ 35%  Fresh: 90%/ <50%  1d. Total: 92%/ 78%  Fresh: 90%/ 61% | Significant increases in total vegetable, fresh vegetable, total fruit and fresh fruit intake.  Significant decrease in intake of french fries.  Females also showed a significantly greater consumption of total vegetables post-intervention. | 1a(i). p<0.001  1a(ii). p<0.001  1b(i). p=0.002  1b(ii). p<0.001  1a(iv). p=0.01  Females 1a(i). p=0.036 |
| Hager et al., 2012* | n=2971 | 1. Dietary outcomes (self-report)  a. Serves of fruits and vegetables (0-3 per month, 1/week, several/week, 1, 2, 3, 4, ≥5/day)  b. Frequency of green salad consumption (0-3 per month, 1/week, several/week, 1, 2, 3, 4, ≥5/day)  c. Frequency of bran or wholegrain cereal consumption (0-3 per month, 1/week, several/week, 1, 2, 3, 4, ≥5/day)  d. Frequency of brown rice or whole wheat product consumption (0-3 per month, 1/week, several/week, 1, 2, 3, 4, ≥5/day) | Mean±SD  (Intervention 1[I1] lecture; Intervention 2[I2] online)  1a. [I1] 4.8±1.5/ 5.1±1.6; [I2] 4.9±1.6/ 4.9±1.7  1b. [I1] 2.5±1.0/ 2.5±1.5; [I2] 2.5±1.0/ 2.5±1.1  1c. [I1] 2.7±1.3/ 3.0±1.4; [I2] 2.7±1.3/ 2.8±1.4  1d. [I1] 2.7±1.3/ 3.1±1.5; [I2] 2.9±1.4/ 3.0±1.5 | Significant differences between groups for change overtime for serves of fruit and vegetables, frequency of bran/wholegrain cereal consumption and brown rice/whole wheat product consumption. | 1.a. p<0.001  1.c. p<0.001  1.d. p<0.001 |
| Hekler et al., 2010* | n=100 | 1. Dietary outcomes – servings/ week  a. Change in vegetable intake  b. Change in fruit intake  c. Change in high-fat dairy intake  d. Change in high-fat meat intake  e. Change in sweet intake  f. Change in processed food intake | Mean±SD  (Intervention 1[I1] food & society course; Intervention 2[I2] comparison courses)  Baseline/ Post-intervention  1a. [I1] 27.9±14.1/ 32.1±17.5; [I2] 27.9±19.3/ 25.5±17.4  1b. [I1] 14.6±7.6/ 16.1±8.6; [I2] 13.0±9.2/ 13.4±9.5  1c. [I1] 8.5±6.5/ 6.3±4.8; [I2] 8.7±6.2/ 8.3±5.8  1d. [I1] 4.0±4.1/ 3.1±4.4; [I2] 4.2±3.7/ 3.8±3.5  1e. [I1] 8.6±5.0/ 7.1±4.6; [I2] 7.7±5.6/ 7.7±5.6  1f. [I1] 5.0±4.2/ 3.6±2.3; [I2] 4.9±4.3/ 4.8±4.2 | Significantly greater increase in vegetable consumption and decrease in high-fat dairy consumption in [I1] compared to [I2].  [I1] showed a significant increase from baseline to post-intervention for vegetable intake and decrease for high-fat dairy intake.  [I2] showed a significant decrease from baseline to post-intervention for vegetable intake. | 1.a. p=0.001  1.c. p=0.02  1.a. p=0.03  1c. p=0.006  1.a. p=0.03 |
| Ince et al., 2008* | n=62 | 1. Health promoting behaviour (self-report; completion of adolescent health promotion scale)  a. Nutrition behaviour | Mean±SD  Baseline/ Post-intervention  1a. 21.2±3.7/ 22.1±3.4 | Significant change in nutrition behaviour | 1.a. p=0.03 |
| Kolodinsky et al., 2008 | n=16 | 1. Food purchase behaviour  a. Number of participants who changed their food purchasing behaviour as a result of intervention | 1a. 4/6 students changes their food purchases as a results of visual food labels. | NR | NR |
| Lachausse., 2012* | n=320 | 1. Fruit and vegetable consumption  a. Change in frequency/day of consumption of fruits  b. Change in frequency/day of consumption of vegetables  2. Self-efficacy  a. Self-efficacy to consume fruits and vegetables | Mean±SD  (Intervention 1[I1] online course; Intervention 2[I2] on-campus course; Comparison group[C])  Baseline/ Post-intervention  1a. [I1] 2.67±1.25/ 3.37±1.60; [I2] 2.93±1.60/ 2.94±1.48;  [C] 3.24±1.55/ 3.15±1.48  1b. [I1] 2.44±1.22/ 2.80±1.35; [I2] 2.64±1.30/ 2.78±1.59;  [C] 2.65±1.32/ 2.80±1.35  2a. [I1] 4.28±0.61/ 4.45±0.54; [I2] 4.3±0.70/ 4.35±0.73;  [C] 4.25±0.63/ 4.31±0.51 | Significant difference between groups for change in frequency of fruit consumption overtime. [I1] significantly increased overtime but [I2] and [C] did not.  Significant difference between groups for change in frequency of vegetable consumption overtime. [I1] significantly increased overtime but [I2] and [C] did not.  Significant difference between groups for fruit and vegetable efficacy overtime. | 1.a. p=0.003  [I1]1.a. p=0.001  1.b. p=0.04  [I1] 1.b. p=0.010  2.a. p=0.04 |
| McClary King et al., 2013* | n=31 | 1. Previous day number of fruit & vegetables  2. Perceived fruit & vegetable planning  3. Perceived health benefits | Mean  (On-campus[ON]; Off-campus[OFF]; Combined[C])  Pre-intervention/Post-intervention  1. [ON]: 3.50/1.14; [OFF]: NR  2. [ON]: 16.38/14.00; [OFF]: NR  3. [C]: 31.57/66.31 | 1, 2. Significant interaction effects  of the intervention and residency. | 1. p<0.05  2. p<0.01 |
| Pearce & Cross, 2013* | n=139 | 1. Dietary recommendation  2. Sources of nutrients  3. Choosing everyday foods  4. Diet disease relationships  5. Nutrition knowledge score | Mean(SD)[Min-Max]  Baseline/After nutrition training *Difference between group means*  1. 6.8(3.1)[1-12]/8.3(3.1)[2-13] *1.5*  2. 35.6(11.2)[12-59]/40.3(8.9)[22-59] *4.7*  3. 4.7(1.7)[1-9]/5.4(1.4)[2-9] *0.6*  4. 9.2(3.7)[2-18]/10.1(3.7)[3-18] *1.0*  5. 56.3(9.7)[33-78]/64.1(7.9)[45-84] *7.8* | 1-3, 5. Significant increase in students’ knowledge of dietary recommendations, sources of nutrients, ability to choose every day foods, and overall nutrition knowledge after course completion. | Difference between group means:  1. p<0.001  2. p=0.001  3. p=0.003  4. p=0.046  5. p<0.001 |
| Pearman et al., 1997* | n=979 | 1. Daily nutrient intake  a. Energy intake (kcal)  b. Fat intake (% of total energy)  c. Protein (% of total energy)  d. Carbohydrate (% of total energy)  e. Calcium intake (mg)  f. Iron (mg)  g. Sodium (mg)  h. Fibre (g)  i. Cholesterol (mg) | Mean  Intervention[I] College A (health course);  Control [C] College B (no health course)  1.a [I] 1710; [C] 1893  1.b[I] 31; [C] 33  1.c [I] 17; [C] 17  1.d [I] 52; [C] 50  1.e [I] 842; [C] 831  1.f [I] 14.1; [C] 13.3  1.g [I] 2775; [C] 2957  1.h [I] 13.0; [C] 13.4  1.i [I] 216; [C] 253 | [I] was significantly more likely to consume fewer calories, less fat, more carbohydrates, less sodium and less cholesterol than [C]. | 1.a. p<0.001  1.b. p<0.01  1.d. p<0.01  1.g. p<0.05  1.i. p<0.0001 |
| Peterson et al., 2010* | n=104 | 1. Change in eating habits (*n,* yes; *n,* no)  a. Eating more fast food  b. Eating more junk food  c. Drinking more soft drinks  d. Eating larger portions  e. Eating one large meal per day  f. Eating less junk food  g. Eating smaller portions  2. Change in food intake (*n,* positive and negative changes in response)  a. Cottage cheese  b. Low-fat salad dressing  c. Deli sandwiches  d. Fresh fruit | Frequency *(n)*  No/ Yes  1.a 99/ 5  1.b 95/ 9  1.c 98/ 6  1.d 96/ 8  1.e 81/ 23  1.f 76/ 28  1.g 88/ 16  Positive change/ Negative change/ No change  2.a 26/ 8/ 67  2.b 34/ 19/ 45  2.c 27/ 51/ 22  2.d 34/ 23/ 46 | 1. Significant improvements for 7 out of 10 eating habits (Data NR for 3 non-significant habits).  2. Significant increase in cottage cheese intake (p<0.001) and low-fat salad dressing intake (p<0.05) and a significant decrease in deli sandwich intake (p<0.01).  No significant change for fresh fruit, steamed vegetables, chicken breast, tossed salad or skim milk (data NR). | 1.a. p=0.000  1.b. p=0.000  1.c. p=0.000  1.d. p=0.000  1.e. p=0.000  1.f. p=0.009  1.g. p=0.000  2.a. p<0.001  2.b. p<0.05  2.c. p<0.01 |
| Reed et al., 2011 | n=278 | 1. Daily cookie intake  a. Chocolate chip  b. M&M  c. Oatmeal  d. Sugar  e. Total  2. Daily fruit intake  a. Apple  b. Banana  c. Orange  d. Pear  e. Total  3. Impact of ‘point of decision’ messages  a. Selected fruit instead of cookie  b. Thought more about food selection  c. Spent more time than usual deciding to select fruit or cookie  d. Selected fruit and cookie  e. Selected cookie | Frequency *(n) –* Average of nine days  Baseline/ Post-intervention  1.a 353/ 362  1.b 257/ 364  1.c 156/ 124  1.d 143/ 198  1.e 909/ 1048  2.a 126/ 138  2.b 171/ 232  2.c 68/ 107  2.d 43/ 50  2.e 408/ 528  Post-intervention  3.a Males: 46.7%; Females: 25.8%  3.b Males: 60.0%; Females: 45.2%  3.c Males: 26.7%; Females: 19.4%  3.d Males: 26.7%; Females: 29.0%  3.e Males: 13.3%; Females: 0.0% | Significant increase in mean fruit intake. | NR |
| Werch et al., 2007* | n=155 | 1. Dietary intake  a. Fruit and vegetable servings (past 7-days)  b. Frequency of consumption of foods containing carbohydrates  c. Frequency of consumption of foods containing fats | Mean±SE  (Intervention 1[I1] Contract; Intervention 2[I2] Consult; Intervention 3[I3] Contract + Consult)  Baseline/ Post-intervention  1.a [I1] 4.62±0.33/ 4.00±0.27; [I2] 3.78±0.33/ 3.86±0.28;  [I3] 4.73±0.33/ 4.29±0.28  1.b [I1] 5.34±0.39/ 4.86±0.34; [I2] 5.37±0.39/ 5.24±0.35;  [I3] 6.02±0.39/ 5.76±0.35  1.c [I1] 3.12±0.32/ 3.40±0.32; [I2] 3.53±0.32/ 4.55±0.32;  [I3] 4.33±0.32/ 4.27±0.32 | Significant increase in consumptions of good fats. | 1.c. p=0.03 |
| Werch et al., 2008 | n=303 | 1. Dietary intake  a. Fruit and vegetable servings (past 7-days)  b. Frequency of consumption of foods containing carbohydrates  c. Frequency of consumption of foods containing fats | Mean±SE  Intervention [I]; Control [C]  Baseline/ Post-intervention  1.a [I] 4.06±0.21/ 4.31±0.17; [C] 3.97±0.20/ 3.73±0.16  1.b [I] 4.55±0.22/ 5.46±0.23; [C] 4.46±0.22/ 4.76±0.23  1.c [I] 3.71±0.22/ 4.34±0.21; [C] 3.54±0.22/ 3.59±0.21 | No significant results. | 1.a. p>0.05  1.b. p>0.05  1.c. p>0.05 |
| Yakusheva et al., 2011 | n=1055 | 1. Restricted food  2. Used weight loss supplement | Mean(SD)  Baseline/follow up  1. 0.54(0.50)/0.47(0.50)  2. 0.05(0.22)/0.07(0.26) | No significance reported | NR |

Weight

| Study | Sample Size | Outcomes | Results | Significance | p-value |
| --- | --- | --- | --- | --- | --- |
| Bowden et al., 2007 | n=108 | 1. Weight outcomes  a. Weight change (lbs) from baseline to follow-up | Mean±SD  (Intervention 1[I1]; Intervention 2[I2]; Intervention 3[I3]; Intervention 4[I4])  Baseline/ Post-intervention  1a. [I1] maintained weight (data NR)  [I2] average 2lb loss at follow-up (data NR)  [I3] average 1.5lb gain at follow-up (data NR)  [I4] average 2lb loss at follow-up (data NR) | No significant difference within or between [I] groups for weight change. | NR |
| Boyle et al., 2011* | n=225 | 1. Body Fat  2. Waist-to-hip-ratio (WHR) | Mean(SD)  Control[C]; Treatment[T]  Pre-test/Post-test  1. Women  [C] 26.14(7.93)/27.27(7.13)  [T] 26.22(7.01)/25.75(7.51)  Men  [C] 12.31(6.22)/12.63(6.51)  [T] 14.99(6.34)/14.23(6.53)  2. Women  [C] 0.75(0.05)/0.77(0.06)  [T] 0.76(0.06)/0.75(0.06)  Men  [C] 0.83(0.07)/0.83(0.06)  [T] 0.85(0.05)/0.85(0.08) | Women in the treatment group had significantly greater reductions in WHR | [W] 2. p<.05 |
| Buscemi et al., 2012 | n=70 | 1. Body mass index  2. Weight | Mean(SD)  Baseline BMI / Follow-up BMI  Group 1[G1]; Group 2[G2]  1. [G1]: 33.43(4.88)/33.61(4.70);  [G2]: 32.26(4.49)/32.56(4.60)  2. [G1]: 202.86(36.10)/203.59(35.97);  [G2]: 189.81(30.98)/191.13(31.30) | No significance | 1. p=0.143 |
| Gow et al., 2010* | n=170 | 1. Weight outcomes  a. BMI (kg/m^2^)  b. Weight change (lbs) from baseline to follow-up | Mean±SD  (Intervention 1[I1]; Intervention 2[I2]; Intervention 3[I3]; Control[C])  Baseline/ Post-intervention  1.a [I1] 23.64±5.18/ 23.39±5.14;  [I2] 25.02±5.41/ 25.21±5.40; [I3] 24.70±4.93/ 24.91±4.94  [C] 24.12±4.72/ 24.30±4.68  1.b. [I1] -0.12±2.92; [I2] 1.47±3.22; [I3] 1.20±2.55  [C] 1.04±3.45 | Significant difference in post BMI between [I1] and the other 3 groups. | 1.a. p<0.05 |
| Hager et al., 2012 | n=2971 | 1. Weight outcomes (self-report)  a. BMI (kg/m^2^)  b. Body mass (kg) | Mean±SD  (Intervention 1[I1] lecture; Intervention 2[I2] online)  Baseline/ Post-intervention  1a. [I1] 23.1±4.0/ 23.1±3.8; [I2] 23.1±4.1/ 23.2±4.0  1b. [I1] 69.5±14.5/ 69.4±14.2; [I2] 68.8±15.2/ 68.9±14.7 | NR | NR |
| Harvey-Berino et al., 2012* | n=336 | 1. Weight loss | Mean(SD)  Baseline BMI (kg/m²)/Post-intervention BMI (kg/m²)  1. BMI<25: 23.3 ± 1.1/22.8 ± 1.3  BMI ≥ 25: 31.0 ± 5.3/30.0 ± 5.2 | All weight changes and healthy lifestyle changes significant | 1. p=.001 |
| Lachausse., 2012 | n=320 | 1. BMI (kg/m^2^)  a. Change in BMI | Mean±SD  (Intervention 1[I1] online course; Intervention 2[I2] on-campus course; Comparison group[C])  Baseline/ Post-intervention  1a. [I1] 29.50±8.78/ 28.75±8.21;  [I2] 29.19±6.64/ 29.00±6.46; [C] 28.31±8.30/ 27.99± 8.13 | No significance | 1.a. p>0.05 |
| LeCheminant et al., 2011 | n=46 | 1. Weight (lbs)  2. Waist (inches)  3. Body Fat (%) | Mean±SD  Control[C]; Pedometer[P]  September/April/change  1. [C]: 146.6±29.1/ 146.9±30.6/ 0.2±7.5  [P]: 148.8±24.9/ 151.3±25.8/ 2.2±5.5  2. [C]: 29.2±4.5/ 29.5±4.1/ 0.2±1.7  [P]: 29.1±3.0/ 29.3±3.1/ 0.2±1.0  3. [C]: 30.9±9.7/ 31.8±9.5/ 0.9±3.1  [P]: 26.4±10.7/ 27.9±10.7/ 1.5±2.7 | No significance | 1. p>0.05  2. p>0.05  3. p>0.05 |
| Musgrave & Thornbury., 1976 | n=14 | 1. Weight loss | Group Total: Entry/final (kg)  1. 75.64kg/71.41kg | No significance reported | NR |
| Tully & Cupples., 2011 | n=12 | 1. Weight (kg)  2. BMI (kg/m²)  3. WHR (cm) | Mean(SD)  Pre-intervention/Post-intervention  10,000 step group[SG]; Control group[CG]  1. [SG]: 64.31(17.35)/63.50(17.11);  [CG]: 67.00(13.77)/67.25(11.76)  2. [SG]: 23.66(6.55)/23.37(6.50);  [CG]: 23.61(3.13)/23.73(2.30)  3. [SG]: 0.83(0.04)/0.82(0.11);  [CG]: 0.83(0.04)/0.83(0.04) | No significant differences | [SG]; [CG]  1. p=0.22; p=0.90  2. p=0.24; p=0.87  3. p=0.25; p=0.11 |
| Wadsworth et al., 2010 | n=91 | 1. Percent body fat | Mean±SD  Intervention [I]; Control [C]  Baseline/ 6 months  1. [I] 35.75±7.02/ 35.68±7.70; [C] 36.86±7.44/ 35.74±7.10 | No significance | 1. p=0.919 |
| Yakusheva et al., 2011* | n=1055 | 1. Weight change (lbs)  2. Proportion trying to lose weight  3. Frequency of exercising outside | Mean(SD)  Baseline/follow up  1. +1.65±8.07  2. 69% / 52%  3. 2.5 times/week / 1 time/week | 1. Significant increase  2. Significant decrease  3. Significant decrease  As weight increased, proportion trying to lose weight decreased as did frequency of exercising. | 1. p<0.05  2. p<0.001  3. p<0.001 |

**Notes to table:** * indicates a significant result within the study named.
